# Supplementary material for: Polymorphisms in ACE, ACE2, AGTR1 genes and severity of COVID-19 disease
Source: PLoS One. 2022 Feb 4;17(2):e0263140. doi: 10.1371/journal.pone.0263140 (PMC8815985; doi:10.1371/journal.pone.0263140)
Supplement: S1 Table — (DOCX) [file pone.0263140.s001.docx]

**S1 Table**. Exact test for Hardy–Weinberg equilibrium (p-value).

|  | **ACE** |  | **ACE2 female (n=119)** | | | |  | **AGTR1** | | |
| --- | --- | --- | --- | --- | --- | --- | --- | --- | --- | --- |
|  | **ID** |  | **rs2074192** | **rs1978124** | **rs2074809** | **rs2074666** |  | **rs5183** | **rs5185** | **rs5186** |
| Allsubjects (n=318) | <0.05 |  | 1 | 0.24 | 0.077 | 0.076 |  | 1 | 1 | 0.68 |
| Outpatients (n=104) | 0.13 |  | 0.16 | 0.73 | 0.55 | 1 |  | 1 | 1 | 1 |
| Hospitalized (n=214) | <0.05 |  | 0.35 | 0.06 | 0.1 | 0.051 |  | 1 | 1 | 0.6 |
