# Supplementary material for: Polymorphisms in ACE, ACE2, AGTR1 genes and severity of COVID-19 disease
Source: PLoS One. 2022 Feb 4;17(2):e0263140. doi: 10.1371/journal.pone.0263140 (PMC8815985; doi:10.1371/journal.pone.0263140)
Supplement: S2 Table — (DOCX) [file pone.0263140.s002.docx]

**S2 Table**. **Genotype and allele frequencies of ACE, ACE2 and AGTR1 SNPs in Outpatients and hospitalized Covid-19 cases, and genotype- and allele type-specific risks**.

| Locus | Model | Genotype | Outpatients | Hospitalized | Odds Ratio | p-value |
| --- | --- | --- | --- | --- | --- | --- |
| **ACE ID (n=309 adjusted by age + gender + comorbidities)** | | | | | | |
|  | Codominant | D/D | 44 (45.8%) | 95 (44.6%) | 1 | 0.57 |
|  |  | I/D | 36 (37.5%) | 74 (34.7%) | 1.07 (0.60-1.91) |  |
|  |  | I/I | 16 (16.7%) | 44 (20.7%) | 0.73 (0.35-1.50) |  |
|  | Dominant | D/D | 44 (45.8%) | 95 (44.6%) | 1 | 0.81 |
|  |  | I/D-I/I | 52 (54.2%) | 118 (55.4%) | 0.94 (0.56-1.58) |  |
|  | Recessive | D/D-I/D | 80 (83.3%) | 169 (79.3%) | 1 | 0.3 |
|  |  | I/I | 16 (16.7%) | 44 (20.7%) | 0.71 (0.36-1.38) |  |
|  | Overdominant | D/D-I/I | 60 (62.5%) | 139 (65.3%) | 1 | 0.55 |
|  |  | I/D | 36 (37.5%) | 74 (34.7%) | 1.18 (0.69-2.02) |  |
|  | Log-additive |  | | | 1.06 (0.75-1.50) | 0.49 |
| **ACE2 rs2074192 (n=309, adjusted by age + gender+ comorbidities)** | | | | | | |
|  | Codominant | G/G | 41 (42.7%) | 111 (52.1%) | 1.00 | 0.083 |
|  |  | G/A | 31 (32.3%) | 32 (15%) | 0.40 (0.16-0.96) |  |
|  |  | A/A | 24 (25%) | 70 (32.9%) | 1.02 (0.55-1.91) |  |
|  | Dominant | G/G | 41 (42.7%) | 111 (52.1%) | 1.00 | 0.42 |
|  |  | G/A-A/A | 55 (57.3%) | 102 (47.9%) | 0.79 (0.45-1.39) |  |
|  | Recessive | G/G-G/A | 72 (75%) | 143 (67.1%) | 1.00 | 0.46 |
|  |  | A/A | 24 (25%) | 70 (32.9%) | 1.25 (0.69-2.25) |  |
|  | Overdominant | G/G-A/A | 65 (67.7%) | 181 (85%) | 1.00 | **0.026** |
|  |  | G/A | 31 (32.3%) | 32 (15%) | 0.39 (0.17-0.91) |  |
|  | Log-additive |  | | | 0.99 (0.73-1.34) | 0.96 |
| **ACE2 rs1978124 (n=309, adjusted by age + gender+ comorbidities)** | | | | | | |
|  | Codominant | G/G | 39 (40.6%) | 102 (47.9%) | 1.00 | 0.17 |
|  |  | A/G | 24 (25%) | 28 (13.2%) | 0.44 (0.19-1.04) |  |
|  |  | A/A | 33 (34.4%) | 83 (39%) | 0.85 (0.47-1.54) |  |
|  | Dominant | G/G | 39 (40.6%) | 102 (47.9%) | 1.00 | 0.23 |
|  |  | A/G- A/A | 57 (59.4%) | 111 (52.4%) | 0.72 (0.42-1.23) |  |
|  | Recessive | G/G -A/G | 63 (65.6%) | 130 (61%) | 1.00 | 0.97 |
|  |  | A/A | 33 (34.4%) | 83 (39%) | 0.99 (0.56-1.74) |  |
|  | Overdominant | A/A-G/G | 72 (75%) | 185 (86.8%) | 1.00 | 0.072 |
|  |  | A/G | 24 (25%) | 28 (13.2%) | 0.47 (0.20-1.08) |  |
|  | Log-additive |  | | | 0.90 (0.68-1.21) | 0.5 |
| **ACE2** **rs2106809 (n=309, adjusted by age + gender + comorbidities)** | | | | | | |
|  | Codominant | T/T | 77 (80.2%) | 148 (69.5%) | 1.00 | 0.11 |
|  |  | T/C | 11 (10.4%) | 21 (9.9%) | 1.68 (0.64-4.41) |  |
|  |  | C/C | 9 (9.4%) | 44 (20.7%) | 2.12 (0.94-4.76) |  |
|  | Dominant | T/T | 77 (80.2%) | 148 (69.5%) | 1.00 | **0.038** |
|  |  | T/C-C/C | 19 (19.8%) | 65 (30.5%) | 1.93 (1.02-3.67) |  |
|  | Recessive | T/T-T/C | 87 (90.6%) | 169 (79.3%) | 1.00 | 0.069 |
|  |  | C/C | 9 (9.4%) | 44 (20.7%) | 2.04 (0.91-4.57) |  |
|  | Overdominant | T/T-C/C | 86 (89.6%) | 192 (90.1%) | 1.00 | 0.37 |
|  |  | T/C | 10 (10.4%) | 21 (9.9%) | 1.55 (0.59-4.06) |  |
|  | Log-additive |  | | | 1.48 (1.01-2.18) | **0.037** |
| **ACE2 rs2285666 (n=309, adjusted by age + gender+ comorbidities)** | | | | | | |
|  | Codominant | G/G | 77 (80.2%) | 154 (72.3%) | 1.00 | 0.19 |
|  |  | G/A | 11 (11.5%) | 20 (9.4%) | 1.19 (0.46-3.08) |  |
|  |  | A/A | 8 (8.3%) | 39 (18.3%) | 2.10 (0.90-4.88) |  |
|  | Dominant | G/G | 77 (80.2%) | 154 (72.3%) | 1.00 | 0.12 |
|  |  | G/A-A/A | 19 (19.8%) | 59 (27.7%) | 1.66 (0.87-3.15) |  |
|  | Recessive | G/G-G/A | 88 (91.7%) | 174 (81.7%) | 1.00 | 0.076 |
|  |  | A/A | 8 (8.3%) | 39 (18.3%) | 2.07 (0.89-4.81) |  |
|  | Overdominant | G/G-A/A | 85 (88.5%) | 193 (90.6%) | 1.00 | 0.83 |
|  |  | G/A | 11 (11.5%) | 20 (9.4%) | 1.11 (0.43-2.85) |  |
|  | Log-additive |  | | | 1.41 (0.95-2.09) | 0.076 |
| **AGTR1 rs5183** (**n=309 adjusted by age + gender + comorbidities)** | | | | | | |
|  | Codominant | A/A | 87 (90.6%) | 186 (87.3%) | 1.00 | 0.44 |
|  |  | A/G | 9 (9.4%) | 26 (12.2%) | 1.17 (0.48-2.81) |  |
|  |  | G/G | 0 (0%) | 1 (0.5%) | NA (0.00-NA) |  |
|  | Dominant | A/A | 87 (90.6%) | 186 (87.3%) | 1.00 | 0.58 |
|  |  | A/G-G/G | 9 (9.4%) | 27 (12.7%) | 1.27 (0.53-3.05) |  |
|  | Recessive | A/A-A/G | 96 (100%) | 212 (99.5%) | 1.00 | 0.22 |
|  |  | G/G | 0 (0%) | 1 (0.5%) | NA (0.00-NA) |  |
|  | Overdominant | A/A-G/G | 87 (90.6%) | 187 (87.8%) | 1.00 | 0.75 |
|  |  | A/G | 9 (9.4%) | 26 (12.2%) | 1.15 (0.48-2.78) |  |
|  | Log-additive |  | | | 1.36 (0.59-3.11) | 0.46 |
| **AGTR1 rs5185(n=309, adjusted by age + gender + comorbidities)** | | | | | | |
|  | --- | T/T | 95 (99.0%) | 210 (98.6%) | 1.00 | 0.71 |
|  |  | T/G | 1 (1.1%) | 3 (1.4%) | 1.56 (0.14-17.28) |  |
| **AGTR1 rs5186 (n=309, adjusted by age + gender + comorbidities)** | | | | | | |
|  | Codominant | A/A | 43 (44.8%) | 114 (53.5%) | 1.00 | 0.29 |
|  |  | A/C | 43 (44.8%) | 86 (40.4%) | 0.74 (0.42-1.27) |  |
|  | Dominant | C/C | 10 (10.4%) | 13 (6.1%) | 0.59 (0.22-1.58) |  |
|  |  | A/A | 43 (44.8%) | 114 (53.5%) | 1.00 | 0.15 |
|  |  | A/C-C/C | 53 (55.2%) | 99 (46.5%) | 0.71 (0.42-1.20) |  |
|  | Recessive | A/A-A/C | 86 (89.6%) | 200 (93.9%) | 1.00 | 0.31 |
|  |  | C/C | 10 (10.4%) | 13 (6.1%) | 0.67 (0.26-1.76) |  |
|  | Overdominant | A/A-C/C | 53 (55.2%) | 127 (59.6%) | 1.00 | 0.36 |
|  |  | A/C | 43 (44.8%) | 86 (40.4%) | 0.79 (0.46-1.34) |  |
|  | Log-additive |  | | | 0.75 (0.50-1.14) | 0.12 |

OR, odds ratio; CI, confidence interval; SNPs, single nucleotide polymorphisms
