# Supplementary material for: Polymorphisms in ACE, ACE2, AGTR1 genes and severity of COVID-19 disease
Source: PLoS One. 2022 Feb 4;17(2):e0263140. doi: 10.1371/journal.pone.0263140 (PMC8815985; doi:10.1371/journal.pone.0263140)
Supplement: S3 Table — (DOCX) [file pone.0263140.s003.docx]

**S3 Table**. Genotype and allele frequencies of ACE2, AGTR1 and ACE SNPs in outpatients and ICU+deceased Covid-19 cases, and genotype- and allele type-specific risks.

| Locus | Model | Genotype | Outpatients | ICU+deceased | | Odds Ratio | p-value | |
| --- | --- | --- | --- | --- | --- | --- | --- | --- |
| **ACE2 FEMALE** (adjusted by age + comorbidities) | | | | | | | | |
| **rs2074192** (n=88) | | | | | | | | |
|  | Codominant | G/G | 8 (18.2%) | 15 (34.1%) | 1.00 | | | 0.048 |
|  |  | G/A | 30 (68.2%) | 18 (40.9%) | 0.27 (0.08-0.86) | | |  |
|  |  | A/A | 6 (13.6%) | 11 (25%) | 0.70 (0.17-2.93) | | |  |
|  | Dominant | G/G | 8 (18.2%) | 15 (34.1%) | 1.00 | | | 0.053 |
|  |  | G/A-A/A | 36 (81.8%) | 29 (65.9%) | 0.35 (0.11-1.04) | | |  |
|  | Recessive | G/G-G/A | 38 (86.4%) | 33 (75%) | 1.00 | | | 0.37 |
|  |  | A/A | 6 (13.6%) | 11 (25%) | 1.70 (0.53-5.53) | | |  |
|  | Overdominant | G/G-A/A | 14 (31.8%) | 26 (59.1%) | 1.00 | | | **0.016** |
|  |  | G/A | 30 (68.2%) | 18 (40.9%) | 0.32 (0.12-0.82) | | |  |
|  | Log-additive |  | | | 0.78 (0.39-1.54) | | | 0.47 |
| **rs1978124** (n=88) | | | | | | | | |
|  | Codominant | G/G | 12 (27.9%) | 19 (43.2%) | 1.00 | | | 0.099 |
|  |  | A/G | 23 (53.5%) | 14 (31.8%) | 0.32 (0.11-0.95) | | |  |
|  |  | A/A | 8 (18.6%) | 11 (25%) | 0.69 (0.19-2.57) | | |  |
|  | Dominant | G/G | 12 (27.9%) | 19 (43.2%) | 1.00 | | | 0.076 |
|  |  | A/G- A/A | 31 (72.1%) | 25 (56.8%) | 0.41 (0.15-1.12) | | |  |
|  | Recessive | G/G -A/G | 35 (81.4%) | 33 (75%) | 1.00 | | | 0.63 |
|  |  | A/A | 8 (18.6%) | 11 (25%) | 1.32 (0.43-4.13) | | |  |
|  | Overdominant | A/A-G/G | 20 (46.5%) | 30 (68.2%) | 1.00 | | | **0.038** |
|  |  | A/G | 23 (53.5%) | 14 (31.8%) | 0.37 (0.14-0.96) | | |  |
|  | Log-additive |  | | | 0.75 (0.40-1.43) | | | 0.38 |
| **rs2106809** (n=88) | | | | | | | | |
|  | Codominant | T/T | 33 (76.7%) | 28 (63.6%) | 1.00 | | | **0.04** |
|  |  | T/C | 9 (20.9%) | 9 (20.4%) | 1.31 (0.41-4.19) | | |  |
|  |  | C/C | 1 (2.3%) | 7 (15.9%) | 12.28 (1.18-128.37) | | |  |
|  | Dominant | T/T | 33 (76.7%) | 28 (63.6%) | 1.00 | | | 0.13 |
|  |  | T/C-C/C | 10 (23.3%) | 16 (36.4%) | 2.21 (0.78-6.28) | | |  |
|  | Recessive | T/T-T/C | 42 (97.7%) | 37 (84.1%) | 1.00 | | | **0.012** |
|  |  | C/C | 1 (2.3%) | 7 (15.9%) | 11.41 (1.12-115.91) | | |  |
|  | Overdominant | T/T-C/C | 34 (79.1%) | 35 (79.5%) | 1.00 | | | 1 |
|  |  | T/C | 9 (20.9%) | 9 (20.4%) | 1.00 (0.32-3.11) | | |  |
|  | Log-additive |  | | | 2.30 (1.04-5.10) | | | **0.03** |
| **rs2285666** (n=88) | | | | | | | | |
|  | Codominant | G/G | 33 (75%) | 25 (56.8%) | 1.00 | | | **0.022** |
|  |  | G/A | 10 (22.7%) | 12 (27.3%) | 1.58 (0.53-4.69) | | |  |
|  |  | A/A | 1 (2.3%) | 7 (15.9%) | 14.43 (1.40-148.29) | | |  |
|  | Dominant | G/G | 33 (75%) | 25 (56.8%) | 1.00 | | | **0.064** |
|  |  | G/A-A/A | 11 (25%) | 19 (43.2%) | 2.51 (0.93-6.80) | | |  |
|  | Recessive | G/G-G/A | 43 (97.7%) | 37 (84.1%) | 1.00 | | | **0.0081** |
|  |  | A/A | 1 (2.3%) | 7 (15.9%) | 12.61 (1.26-125.87) | | |  |
|  | Overdominant | G/G-A/A | 34 (77.3%) | 32 (72.7%) | 1.00 | | | 0.75 |
|  |  | G/A | 10 (22.7%) | 12 (27.3%) | 1.19 (0.41-3.40) | | |  |
|  | Log-additive |  | | | 2.53 (1.16-5.50) | | | **0.013** |
| **ACE2 MALE** (adjusted by age + comorbidities) | | | | | | | | |
| **rs2074192** (n=147) | | | | | | | | |
|  |  | G/G | 33 (64.7%) | 63 (65.6%) | 1.00 | | | 0.91 |
|  |  | A/A | 18 (35.3%) | 33 (34.4%) | 0.96 (0.43-2.10) | | |  |
| **rs1978124** (n=147) | | | | | | | | |
|  |  | G/G | 26 (51%) | 50 (52.1%) | 1.00 | | | 0.68 |
|  |  | A/A | 25 (49%) | 46 (47.9%) | 0.86 (0.41-1.81) | | |  |
| **rs2106809** (n=147) | | | | | | | | |
|  |  | T/T | 43 (84.3%) | 70 (72.9%) | 1.00 | | | 0.16 |
|  |  | C/C | 8 (15.7%) | 26 (27.1%) | 1.93 (0.75-4.96) | | |  |
| **rs2285666** (n=147) | | | | | | | | |
|  |  | G/G | 44 (86.3%) | 73 (76%) | 1.00 | | | 0.22 |
|  |  | A/A | 7 (13.7%) | 23 (24%) | 1.82 (0.68-4.86) | | |  |
| **AGTR1** (n=235 adjusted by age + gender + comorbidities) | | | | | | | | |
| **rs5183** | | | | | | | | |
|  |  | A/A | 87 (90.6%) | 122 (87.8%) | 1.00 | | | 0.67 |
|  |  | A/G | 9 (9.4%) | 17 (12.2%) | 1.24 (0.46-3.32) | | |  |
| **rs5185** | | | | | | | | |
|  | --- | T/T | 95 (99%) | 138 (99.3%) | 1.00 | | | 0.83 |
|  |  | T/G | 1 (1.1%) | 1 (0.7%) | 0.71 (0.03-17.17) | | |  |
| **rs5186** | | | | | | | | |
|  | Codominant | A/A | 43 (44.8%) | 74 (53.2%) | 1.00 | | | 0.38 |
|  |  | A/C | 43 (44.8%) | 55 (39.6%) | 0.70 (0.38-1.29) | | |  |
|  |  | C/C | 10 (10.4%) | 10 (7.2%) | 0.57 (0.20-1.64) | | |  |
|  | Dominant | A/A | 43 (44.8%) | 74 (53.2%) | 1.00 | | | 0.18 |
|  |  | A/C-C/C | 53 (55.2%) | 65 (46.8%) | 0.67 (0.37-1.21) | | |  |
|  | Recessive | A/A-A/C | 86 (90.5%) | 129 (92.8%) | 1.00 | | | 0.44 |
|  |  | C/C | 10 (10.4%) | 10 (7.2%) | 0.67 (0.24-1.86) | | |  |
|  | Overdominant | A/A-C/C | 53 (55.2%) | 84 (60.4%) | 1.00 | | | 0.36 |
|  |  | A/C | 43 (44.8%) | 55 (39.6%) | 0.76 (0.42-1.37) | | |  |
|  | Log-additive |  | | | 0.73 (0.47-1.15) | | | 0.17 |
| **ACE ID** (n=235 adjusted by age + gender + comorbidities) | | | | | | | | |
|  | Codominant | D/D | 42 (44.7%) | 66 (47.1%) | 1 | | | 0.63 |
|  |  | I/D | 36 (38.3%) | 48 (34.3%) | 0.77 (0.40-1.46) | | |  |
|  |  | I/I | 16 (17%) | 26 (18.6%) | 1.09 (0.48-2.47) | | |  |
|  | Dominant | D/D | 42 (44.7%) | 66 (47.1%) | 1 | | | 0.61 |
|  |  | I/D-I/I | 52 (55.3%) | 74 (52.9%) | 0.86 (0.48-1.54) | | |  |
|  | Recessive | D/D-I/D | 78 (83%) | 114 (81.4%) | 1 | | | 0.6 |
|  |  | I/I | 16 (17%) | 26 (18.6%) | 1.23 (0.57-2.63) | | |  |
|  | Overdominant | D/D-I/I | 58 (61.7%) | 92 (65.7%) | 1 | | | 0.34 |
|  |  | I/D | 36 (38.3%) | 48 (34.3%) | 0.75 (0.41-1.37) | | |  |
|  | Log-additive |  | | | 0.99 (0.67-1.45) | | | 0.94 |

OR, odds ratio; CI, confidence interval; SNPs, single nucleotide polymorphisms.
