# Supplementary material for: Polymorphisms in ACE, ACE2, AGTR1 genes and severity of COVID-19 disease
Source: PLoS One. 2022 Feb 4;17(2):e0263140. doi: 10.1371/journal.pone.0263140 (PMC8815985; doi:10.1371/journal.pone.0263140)
Supplement: S4 Table — Interaction analysis with comorbidities and different SNPs. (DOCX) [file pone.0263140.s004.docx]

**S4 Table. Relationship of comorbidities with the different SNPs in COVID-19 patients.** Interaction analysis with comorbidities and different SNPs.

|  | **NO COMORBIDITIES** | | |  | **COMORBIDITIES** | | | | | | **OR (95% CI)** | | |
| --- | --- | --- | --- | --- | --- | --- | --- | --- | --- | --- | --- | --- | --- |
|  | **Outpatients**  **(n=104)** | | **Hospitalized (n=214)** | **OR (95% CI)** | **Outpatients**  **(n=104)** | | | **Hospitalized (n=214)** | | |  |  |  |
| **ACE2 FEMALE (n= 119, adjusted by age)** | | | | | | | | | | | | | |
| **rs2074192** | | | | | | | | | | | | | |
| **G/G** | | 4 | 13 | 1.00 | | 4 | | 11 | 0.52 (0.09-2.85) | | | | |
| **G/A*** | | 27 | 13 | 0.13 (0.03-0.52) | | 3 | | 19 | 1.27 (0.22-7.19) | | | | |
| **A/A** | | 4 | 9 | 0.61 (0.12-3.26) | | 2 | | 9 | 0.73 (0.10-5.52) | | | | |
|  | |  |  |  | |  | | **Interaction p-value: 0.019** | | | | | |
| **rs1978124** | | | | | | | | | | | | | |
| **G/G** | | 9 | 13 | 1.00 | | 3 | | 15 | | 2.60 (0.56-12.14) | | | |
| **A/G** | | 18 | 13 | 0.44 (0.14-1.39) | | 5 | | 15 | | 1.15 (0.27-4.85) | | | |
| **A/A** | | 7 | 9 | 0.73 (0.19-2.87) | | 1 | | 9 | | 3.16 (0.31-32.37) | | | |
|  | | |  |  | |  | | Interaction p-value: 0.92 | | | | | |
| **rs2106809** | | | | | | | | | | | | | |
| **T/T** | | 27 | 23 | 1.00 | | 6 | | 22 | | 2.63 (0.84-8.27) | | | |
| **T/C** | | 7 | 8 | 1.56 (0.47-5.16) | | 2 | | 13 | | 5.80 (1.14-29.56) | | | |
| **C/C** | | 0 | 4 | --- | | 1 | | 4 | | 4.35 (0.44-42.82) | | | |
|  | | |  |  | |  | | Interaction p-value: 0.22 | | | | | |
| **rs2285666** | | | | | | | | | | | | | |
| **G/G** | | 27 | 22 | 1.00 | | | 6 | 24 | | 3.22 (1.05-9.93) | | | |
| **G/A** | | 8 | 8 | 1.19 (0.37-3.80) | | | 2 | 12 | | 4.73 (0.90-24.84) | | | |
| **A/A** | | 0 | 5 | --- | | | 1 | 3 | | 3.12 (0.29-33.49) | | | |
|  | | |  |  | | |  | Interaction p-value: 0.15 | | | | | |
| **ACE2 MALE (n=190, adjusted by age)** | | | | | | | | | | | | | |
| **rs2074192** | | | | | | | | | | | | | |
| **G/G** | | 24 | 43 | 1.00 | | 9 | | 44 | 1.81 (0.72-4.56) | | | | |
| **A/A** | | 13 | 21 | 1.00 (0.41-2.41) | | 5 | | 31 | 2.17 (0.70-6.68) | | | | |
|  | |  |  |  | |  | | Interaction p-value: 0.81 | | | | | |
| **rs1978124** | | | | | | | | | | | | | |
| **G/G** | | 20 | 33 | 1.00 | | 6 | | 41 | 2.48 (0.84-7.31) | | | | |
| **A/A** | | 17 | 31 | 1.02 (0.44-2.36) | | 8 | | 34 | 1.57 (0.57-4.33) | | | | |
|  | |  |  |  | |  | | Interaction p-value: 0.51 | | | | | |
| **rs2106809** | | | | | | | | | | | | | |
| **T/T** | | 32 | 49 | 1.00 | | 11 | | 54 | 1.89 (0.80-4.48) | | | | |
| **C/C** | | 5 | 15 | 1.71 (0.55-5.31) | | 3 | | 21 | 3.09 (0.82-11.67) | | | | |
|  | |  |  |  | |  | | Interaction p-value: 0.96 | | | | | |
| **rs2285666** | | | | | | | | | | | | | |
| **G/G** | | 33 | 51 | 1.00 | | 11 | | 57 | 2.01 (0.85-4.71) | | | | |
| **A/A** | | 4 | 13 | 1.87 (0.55-6.39) | | 3 | | 18 | 2.66 (0.69-10.16) | | | | |
|  | |  |  |  | |  | | Interaction p-value: 0.72 | | | | | |
| **AGTR1 (n=309, adjusted by age and gender)** | | | | | | | | | | | | | |
| **rs5183** | | |  |  | |  | |  |  | | | |  |
| **A/A*** | | 65 | 88 | 1.00 | | 22 | | 98 | 2.22 (1.21-4.06) | | | | |
| **A/G** | | 7 | 10 | 1.18 (0.41-3.46) | | 2 | | 16 | 2.80 (0.59-13.28) | | | | |
| **G/G** | | 0 | 1 | --- | | 0 | | 0 | --- | | | | |
|  | | |  |  | |  | | Interaction p-value: 0.99 | | | | | |
| **rs5185** | | |  |  | |  | |  |  | | |  | |
| **T/T** | | 72 | 97 | 1.00 | | 23 | | 113 | 2.30 (1.27-4.15) | | | | |
| **T/G** | | 0 | 2 | --- | | 1 | | 1 | 0.50 (0.03-8.33) | | | | |
|  | | |  |  | |  | | Interaction p-value: 0.058 | | | | | |
| **rs5186** | | |  |  | |  | |  |  | | |  | |
| **A/A** | | 32 | 48 | 1.00 | | 11 | | 66 | 2.23 (0.96-5.15) | | | | |
| **A/C** | | 34 | 46 | 0.72 (0.37-1.40) | | 9 | | 40 | 1.57 (0.62-3.94) | | | | |
| **C/C** | | 6 | 5 | 0.57 (0.15-2.18) | | 4 | | 8 | 1.07 (0.28-4.11) | | | | |
|  | | |  |  | |  | | Interaction p-value: 0.98 | | | | | |
| **ACE (n=309, adjusted by age and gender)** | | | | | | | | | | | | | |
| **D/D*** | | 34 | 44 | 1.00 | | 10 | | 51 | 2.51 (1.06-5.94) | | | | |
| **I/D** | | 27 | 33 | 0.95 (0.47-1.94) | | 9 | | 41 | 2.23 (0.91-5.44) | | | | |
| **I/I** | | 11 | 22 | 1.69 (0.70-4.08) | | 5 | | 22 | 2.28 (0.75-6.92) | | | | |
|  | | |  |  | |  | | Interaction p-value: 0.71 | | | | | |

*Significantly differences (p< 0.0001).
