# Supplementary material for: Polymorphisms in ACE, ACE2, AGTR1 genes and severity of COVID-19 disease
Source: PLoS One. 2022 Feb 4;17(2):e0263140. doi: 10.1371/journal.pone.0263140 (PMC8815985; doi:10.1371/journal.pone.0263140)
Supplement: S5 Table — Interaction analysis with comorbidities and different SNPs. (DOCX) [file pone.0263140.s005.docx]

**S5 Table. Relationship of comorbidities with the different SNPs in outpatients and ICU+deceased COVID-19 patients.**

Interaction analysis with comorbidities and different SNPs.

|  | **NO COMORBIDITIES** | |  | | **COMORBIDITIES** | | |  | |
| --- | --- | --- | --- | --- | --- | --- | --- | --- | --- |
|  | **Outpatients** | **ICU+deceased** | | **OR (95% CI)** | **Outpatients** | **ICU+deceased** | | | **OR (95% CI)** |
| **ACE2 FEMALE (n=88, adjusted by age)** | | | | | | | | | |
| **rs2074192** | | |  | |  |  | |  | |
| **G/G** | 4 | 10 | 1.00 | | 4 | 5 | | 0.22 (0.03-1.59) | |
| **G/A** | 27 | 7 | 0.06 (0.01-0.33) | | 3 | 11 | | 0.66 (0.10-4.47) | |
| **A/A** | 4 | 5 | 0.34 (0.05-2.22) | | 2 | 6 | | 0.44 (0.05-3.99) | |
|  | |  |  | |  | **Interaction p-value: 0.0074** | | | |
| **rs1978124** | | |  | |  |  |  | | |
| **G/G** | 9 | 9 | 1.00 | | 3 | 10 | 2.37 (0.46-12.31) | | |
| **A/G** | 18 | 7 | 0.34 (0.09-1.27) | | 5 | 7 | 0.71 (0.14-3.55) | | |
| **A/A** | 7 | 6 | 0.63 (0.14-2.93) | | 1 | 5 | 2.18 (0.19-25.44) | | |
|  | |  |  | |  | Interaction p-value: 0.94 | | | |
| **rs2106809** | | |  | |  |  |  | | |
| **T/T** | 27 | 14 | 1.00 | | 6 | 14 | 2.69 (0.78-9.23) | | |
| **T/C** | 7 | 4 | 1.36 (0.32-5.81) | | 2 | 5 | 3.29 (0.50-21.61) | | |
| **C/C** | 0 | 4 | --- | | 1 | 3 | 6.12 (0.55-67.69) | | |
|  | |  |  | |  | Interaction p-value: 0.2 | | | |
| **rs2285666** | | |  | |  |  |  | | |
| **G/G** | 27 | 12 | 1.00 | | 6 | 13 | 2.76 (0.77-9.86) | | |
| **G/A** | 8 | 5 | 1.41 (0.36-5.56) | | 2 | 7 | 5.40 (0.90-32.43) | | |
| **A/A** | 0 | 5 | --- | | 1 | 2 | 4.60 (0.35-59.79) | | |
|  | |  |  | |  | Interaction p-value: 0.12 | | | |
| **ACE2 FEMALE (n=147, adjusted by age)** | | | | | | | | | |
| **rs2074192** | | |  | |  |  | |  | |
| **G/G** | 24 | 29 | 1.00 | | 9 | 34 | | 2.23 (0.85-5.86) | |
| **A/A** | 13 | 12 | 0.99 (0.36-2.72) | | 5 | 21 | | 2.03 (0.62-6.64) | |
|  | |  |  | |  | Interaction p-value: 0.92 | | | |
| **rs1978124** | | |  | |  |  |  | | |
| **G/G** | 20 | 20 | 1.00 | | 6 | 30 | 2.84 (0.91-8.93) | | |
| **A/A** | 17 | 21 | 1.04 (0.41-2.68) | | 8 | 25 | 1.75 (0.59-5.20) | | |
|  | |  |  | |  | Interaction p-value: 0.5 | | | |
| **rs2106809** | | |  | |  |  |  | | |
| **T/T** | 32 | 31 | 1.00 | | 11 | 39 | 2.08 (0.84-5.15) | | |
| **C/C** | 5 | 10 | 1.97 (0.57-6.80) | | 3 | 16 | 3.89 (0.98-15.48) | | |
|  | |  |  | |  | Interaction p-value: 0.96 | | | |
| **rs2285666** | | |  | |  |  |  | | |
| **G/G** | 33 | 32 | 1.00 | | 11 | 41 | 2.22 (0.91-5.44) | | |
| **A/A** | 4 | 9 | 2.10 (0.56-7.82) | | 3 | 14 | 3.34 (0.83-13.52) | | |
|  | |  |  | |  | Interaction p-value: 0.74 | | | |
| **AGTR1** **(n=235, adjusted by age and gender)** | | | | | | | | | |
| **rs5183** | |  |  | |  |  | |  | |
| **A/A** | 65 | 56 | 1.00 | | 22 | 66 | | 2.24 (1.17-4.29) | |
| **A/G** | 7 | 6 | 1.25 (0.36-4.39) | | 2 | 11 | | 2.70 (0.54-13.52) | |
|  | |  |  | |  | Interaction p-value: 0.97 | | | |
| **rs5185** | |  |  | |  |  | |  | |
| **T/T** | 72 | 61 | 1.00 | | 23 | 77 | | 2.38 (1.27-4.46) | |
| **T/G** | 0 | 1 | --- | | 1 | 0 | | 0.00 | |
|  | |  |  | |  | Interaction p-value: 0.019 | | | |
| **rs5186** | |  |  | |  |  | |  | |
| **A/A** | 32 | 30 | 1.00 | | 11 | 44 | | 2.10 (0.85-5.20) | |
| **A/C** | 34 | 29 | 0.69 (0.32-1.47) | | 9 | 26 | | 1.48 (0.55-3.99) | |
| **C/C** | 6 | 3 | 0.47 (0.10-2.22) | | 4 | 7 | | 1.44 (0.34-6.06) | |
|  | |  |  | |  | Interaction p-value: 0.94 | | | |
| **ACE (n=235, adjusted by age and gender)** | | | | | | | | | |
| **D/D** | 34 | 29 | 1.00 | | 10 | 36 | | 2.10 (0.85-5.20) | |
| **I/D** | 27 | 19 | 0.81 (0.35-1.85) | | 9 | 29 | | 1.48 (0.55-3.99) | |
| **I/I** | 11 | 14 | 1.81 (0.67-4.92) | | 5 | 12 | | 1.44 (0.34-6.06) | |
|  | |  |  | |  | Interaction p-value: 0.3 | | | |
